# Cancer detection in primary care: insights from general practitioners

**DOI:** 10.1038/bjc.2015.41

**Published:** 2015-03-03

**Authors:** T Green, K Atkin, U Macleod

**Affiliations:** 1 Hull York Medical School, University of Hull, Hertford Building, Cottingham Road, Hull HU6 7RX, UK; 2 Department of Health Sciences, University of York, Seebohm Rowntree Building, Heslington, York YO10 5DD, UK

**Keywords:** GPs, cancer symptoms, recognition and referral, qualitative research

## Abstract

**Background:**

General practitioners (GPs) have a key role in cancer detection as the usual first point of contact for patients with potential cancer symptoms. Nevertheless, there is limited work that investigates their perceptions of their role in the early detection of cancer. To address this gap, we aimed to gain an in-depth understanding of cancer diagnosis from the perspective of GPs.

**Methods:**

Individual face-to-face semi-structured interviews were conducted with 55 GPs from the North and North East of England and Greater London. All interviews were recorded and professionally transcribed verbatim. Repeated reading and co-coding engendered systematic thematic analysis across the interview material.

**Results:**

Three main themes emerged from the analysis of our data. First, we identified the burden of early cancer detection in general practice, both related to the anxiety and symptoms patients bring to GPs and the need for GPs to recognise patterns of cancer symptoms and refer appropriately; second, this burden is intensified by a perceived fragmentation of services within the National Health Service (NHS); and third, it is made more complex by the interface between general practice and public health.

**Conclusions:**

GPs occupy a challenging but pivotal role in cancer detection. It is crucial that this role be supported by policy and research.

Diagnosing cancer when it is at an early stage is important, as the stage of disease at diagnosis is related to survival for many cancers. Because of this, there has been increasing interest in how patients and professionals recognise cancer symptoms (Department of Health, 2007), which has resulted in an increased interest in all stages of the pathway to diagnosis. This pathway is complex; timely diagnosis is dependent on patients identifying a new symptom or something different, interpreting that change as something about which they should see their doctor (Macleod *et al*, 2009), negotiating the system to obtain an appointment and then the general practitioner (GP) recognising the symptoms as a presentation of potential cancer. This pathway is not as linear as initial models of the pathway to diagnosis suggested (Andersen *et al*, 1995). More recent models of the pathway, based on literature review (Walter *et al*, 2012), have suggested that the process of patient appraisal and self-management continues after the decision to consult a health-care professional takes place and interacts with the health-care professional in turn appraising, investigating and referring the patient for further investigation. Central to this process, however, are two parties: the patient and the health-care professional, who in a UK context is most commonly a GP.

In addition, complexity is added in the United Kingdom (and similar health-care systems) owing to the role of the GP in ‘gate-keeping' access to referral to specialist care (Vedsted and Olesen, 2011). The interaction between the GP and the patient can therefore be seen as the front line for contact between public/patient and the National Health Service (NHS) with respect to the understanding of symptoms, presentation and GP response, which Walter *et al* (2012) state form the ‘key determinants of outcomes in cancer'. Primary care is therefore the focus of considerable research and policy activity in the area of cancer symptom recognition and referral. However, although work to date has included a GP perspective in cancer-specific studies and has focussed on cancer detection in primary care, including, for example, the development of algorithms to assist with risk stratification of symptom presentation (Hamilton *et al*, 2013; Hippisley-Cox and Coupland, 2013; Stapley *et al*, 2013; Walker *et al*, 2013), there is limited work that investigates GPs' understanding of patients' awareness of cancer symptoms or on GPs' views of their own role in early detection.

An exception to this is a focus group study and national survey conducted by Daly and Collins (2007) of GPs in Ireland who identified barriers to early diagnosis within their health-care system. Their findings indicated that late diagnosis was related to patient delay, lack of primary care access to diagnostic investigations, barriers to secondary care referral, lack of clarity around cancer screening, poor communication between primary and secondary care and inequity of services for patients who are unable to pay for private care and treatment. Daly and Collins (2007) concluded that, at the time of their study, general practice was not adequately resourced to meet the demands of earlier cancer detection. More recently, Cook *et al* (2014) have studied the views of primary care staff from six practices in the North West of England with regard to the promotion to patients of earlier symptom presentation and the management of risk in cancer detection and referral in primary care. These authors highlighted their respondents' concern at the lack of understanding on the part of policymakers and those implementing cancer initiatives of the impact of ‘inherent uncertainty, organisational change and competing priorities' within primary care. It seems appropriate therefore to further study this group of professionals who are at the front line in cancer detection.

Our overall aim in conducting this study was to obtain an in-depth understanding of cancer diagnosis from the perspective of GPs, and to develop a framework for conceptualising the potential of the GP's role to improve cancer recognition and referral. The objectives included identifying GPs' understanding of their role with respect to cancer awareness, screening and early detection. We also examined attitudes to awareness and early diagnosis, including the extent to which prompt presentation with symptoms is welcomed on the one hand or considered to be a ‘worried well' burden on resources. We also explored participants' understandings their gate-keeping role and its impact on early cancer diagnosis. Further in recognition that cancer detection is not merely about symptom presentation, we explored attitudes to screening and the role of primary care in supporting screening coverage to consider whether there is scope for enhancing the role of primary care in promoting cancer screening coverage.

## Materials and methods

As our aim was to explore how the role of GPs could be maximised with respect to the early diagnosis of cancer, we considered that in-depth semi-structured interviews with GPs were the most appropriate research technique to capture this information. Interviews were conducted by the first author, and the majority were carried out in participants' workplaces. A topic guide was used, and each interview was digitally recorded (with the permission of the interviewee) and professionally transcribed verbatim. The main topics explored were as follows: the GP's role in the early detection of cancer, the role of primary care with respect to cancer awareness, the role of GPs with respect to cancer screening, and NHS changes and policy issues. Each topic entailed a series of open-ended questions and additional probes. GPs' responses were followed up as appropriate (Supplementary Online Material 1). GPs were also asked to describe the demographics of their patient populations. We accessed the National General Practice Profiles (NGPP) for data on size of practice, deprivation score and ethnic make-up (Supplementary Online Material 2). The duration of the interviews ranged between 60 and 75 min. GPs were remunerated for their time.

### Participants

Participants were drawn from 16 former primary care trusts (11: North and North East England; and 5: Greater London). To aid recruitment, colleagues in local Primary Care Research Networks contacted practices on behalf of the research team with information regarding the study. Fifty-five GPs responded and were contacted by the researcher to arrange interview dates and times. Interviews were conducted from May 2012 to April 2013.

### Data analysis

Analysis of the interview data was guided by the research objectives and was ongoing throughout the fieldwork. We adopted an iterative approach, whereby themes were identified and fed back into the data collection process. This method of moving between formulating theory and analysing data facilitated a better understanding of the GP's role in early cancer diagnosis. The transcripts were double-coded (TG, UM) and the research team held regular meetings to discuss the emergent themes from the fieldwork material. Thematic analysis explored relationships between patterns, categories and descriptive themes (Brewer, 2000). To introduce transparency, a systematic approach to data analysis was used, engaging detailed familiarisation, identification and indexing of key themes; contextualising of themes in relation to the framework of cancer diagnosis in primary care; and interpreting them within the context of theoretical themes relevant to the interview material. We then compared across cases by highlighting potential similarities and differences, and finally related these to characteristics of the respondent that could be reasonably justified as an explanation that mediated experience, such as demography of practice (Silverman, 2009). This process involved understanding the meaning of actions, beliefs, attitudes and views from the range and frequency of participants' narratives, as well as consistent cross-referencing, which looked for similarities and differences within the sample. This enabled patterns of views and perceptions to be identified, comparisons made and contradictions to be explored. Data were uploaded to *NVivo* 10, and a coding strategy and coding framework were developed to facilitate data retrieval and comparative analysis. Records of data collection and analysis were kept at all stages.

### Ethics

The study was submitted to the Hull York Medical School Research Ethics Committee for ethical approval and to the relevant NHS R&D departments for research governance approval. The main ethical issues related to confidentiality of participants and to storage of data. We assured our participants of confidentiality, and data have been anonymised accordingly. All data from the fieldwork have been stored securely. Pseudonyms denote chronology of interview/GP gender/NGPP deprivation score.

## Results

Results are presented under the following three principal themes: (1) symptom recognition and referral in general practice; (2) GPs' perceptions of the fragmentation of NHS services and care; and (3) primary care/public health interface. Each principal theme has a series of subthemes; illustrative data are presented in Box 1, Box 2 and Box 3. The themes and subthemes are summarised in Box 4.

### Symptom recognition and referral by GPs

This principal theme relates to how cancer plays a major part in GPs' daily practice. This is owing to public perceptions of cancer that are brought to GPs' attention and discussed during consultations, the recognition and referral of symptoms and barriers to referral.

*Public perceptions of cancer*. GPs were aware that they would identify only a small number of new cancer patients during their professional lifetime. However, interview data indicate that cancer took up a considerable amount of participants' time and resources. Cancer as a potential diagnosis, rather than a disease, was substantial in terms of public concern and, as a result of this, patient anxiety and GP time. Participants across the sample indicated how late presentation of symptoms was often linked to patients' socio-economic and socio-cultural circumstances. This was an issue GPs struggled with because the outcome was often later presentation, referral and diagnosis, and thus higher mortality rates for practices in these areas.

*Recognition of symptoms*. Participants were aware that many symptoms patients presented with that were indicative of cancer would prove to be non-malignant but at the same time felt under pressure not to ‘miss' potential cancer symptoms. Data show the uncertainty that surrounds non-specific symptoms and the skill needed in filtering out patients who might be at risk of cancer from those presenting with self-limiting problems.

*Gatekeeper role*. Participants valued their gatekeeper role and perceived that the GP's skill was to identify those patients in need of further investigation or referral from those who could be managed within primary care. The ability to perform this role adequately was perceived to be dependent on the quality of the GP/patient relationship and the GP's role as patients' advocate. Participants perceived that their role was undergoing change owing to NHS reforms, and that there was some anxiety regarding how the joint responsibilities of patient advocacy and resource management would play out in practice.

*Referral*. Participants relied on guidelines and the 2-week-wait (2WW) urgent referral routes available for potential cancer symptoms in England (National Institute for Health and Clinical Care Excellence (NICE), 2005). Although GPs valued 2WW, they also highlighted its limitations when symptoms do not meet guideline criteria, and referral criteria then acted as a barrier. Several GPs called for a generic route for suspicious symptoms. GPs had strategies to overcome some of the barriers, although this situation was managed more easily when there were opportunities for dialogue with secondary care colleagues. Participants perceived that the primary/secondary care relationship had changed as cancer care at secondary level became more specialised.

### Fragmentation of NHS services and care

Our second principal theme focuses on changes in primary care provision and how these affected GPs' practice with regard to continuity of care and the perceived impact of organisational change on GP practice.

*Continuity of care*. GPs recognised the significance of being the first port of call for patients, and commented that this was key to establishing patients' trust in the GP and primary care more generally. They perceived that the availability of ports of call other than the GP practice affected the provision of continuity of care to patients, and indicated how multiple points of provision could have a negative impact on the detection of cancer symptoms and timeliness of diagnosis.

*Organisational change*. Participants were concerned about changes to the GP remit – for example, the introduction of Clinical Commissioning Groups (CCGs) that include experienced GPs in their consortia. Participants felt that their role as patient advocate was one of the strengths of primary care, and felt that this role might be compromised by recent reorganisation of primary care and increased responsibility for resource management, which they felt might negatively affect referral decisions. GPs also perceived that responsibility for increased administrative tasks would reduce surgery time with patients.

### Primary care/public health interface

In this final section, we contextualise our findings in relation to GPs' perceptions of the wider public health field with regard to cancer screening programmes and awareness-raising initiatives.

*Cancer screening*. Participants were unified in their views about the criteria that screening programmes should fulfil. Although overall support for screening was evident, participants' perceptions of some programmes were affected by conflict among experts in the field, in particular mammography (although not screening as such, testing for prostate cancer also caused anxiety). All participants, however, said that they promoted screening to their patients, and the majority perceived that GP endorsement of screening would increase patient compliance. Participants commented on the challenges of managing patients' responses to, and understandings of, screening.

*Awareness-raising initiatives*. Participants commented positively on awareness-raising campaigns and welcomed attempts to educate the public about the signs and symptoms of cancer. Although the majority of participants were mostly of the view that educating the public about cancer symptoms via campaign initiatives is a worthwhile endeavour, they simultaneously commented on some of the negative consequences of these owing to increased numbers of consultations, time pressures and limited resources. Some GPs also thought that the push to identify symptoms at an earlier stage could result in unforeseen challenges. Several participants noted that public health initiatives had more impact on members of the public already sensitised to health issues, as well as patients who they termed the ‘worried well'. All of our respondents articulated a commitment to patient ‘safety netting' and to educating patients around cancer warning signs and symptoms.

*GP role in raising awareness*. There was agreement across the study sample that awareness-raising initiatives at times had little effect on some members of the public and they gave their views on who these groups and individuals might be. The majority of participants felt that their role in raising awareness was practice-based, that is, they perceived the GP's role in enhancing patients' awareness of the signs and symptoms of cancer would be best achieved within the confines of the surgery and that GP/patient interactions would support public health messages. A small number of participants saw a more proactive role for themselves or for primary care more generally in order to engage patients who, for a variety of reasons, might not be reached by public health initiatives.

## Discussion

The aim of this article was to emphasise the role that GPs have at a time of great upheaval within the NHS because for the most part they are the first port of call for the general public. The strength of this work is that it is the largest in-depth study of GPs' understandings and perceptions of their role in early cancer detection. We have presented findings that relate to all aspects of this task, and we have done so at a time when many initiatives to improve early diagnosis were taking place in England. The limitations relate to the generalisability of qualitative research, and that in seeking to present an overview of our data to provide insight into the breadth of activity, we have been unable to present some of the depth relating to particular aspects of the GP role.

Findings from our study show how the potential diagnosis of cancer was a concern for our participants that absorbed much of their time. It is the case, however, that inherent uncertainties underscore cancer symptom recognition and referral decisions in primary care; the skill required in the management of risk and uncertainty has been noted by others (O'Riordan *et al*, 2011; Round *et al*, 2013; Cook *et al*, 2014). Our participants were aware that the majority of patients who present with potential cancer symptoms will have a diagnosis other than cancer (Macleod *et al*, 2009; Richards, 2009; Jensen *et al*, 2014). Clearly, the detection of cancer in primary care is complex, and it can involve several appointments and investigations (Walter *et al*, 2012). Although all participants welcomed the 2WW criteria, references to ‘gut instinct' were evident across the sample and had a role in GPs' ability, in the absence of red flag symptoms, to identify patients in need of further investigation (to either rule in or rule out cancer).

Stolper *et al* (2010) have commented on the notion of uncertainty in general practice when GPs are faced with ‘complicated, vague problems'. The GPs in our study stressed that patients with vague symptoms, which over time turned out to be those of cancer, were more likely to receive a later diagnosis because their symptoms did not meet urgent referral criteria. GPs also applied a ‘watch and wait' safety-net approach to some patients because they were aware that, as Ely *et al* (2011) attest, in primary care ‘diagnosis often emerges over time' (Walter *et al*, 2012). To ease this situation, GPs called for a generic referral route for suspected cancer symptoms, alongside increased opportunity for communication with secondary care colleagues.

Round *et al* (2013) comment that fragmentation within primary care has occurred because of increased numbers of part-time and salaried GPs and the use of locum cover. They identified this as a root cause of the demise of continuity of care because of the ‘difficulty in seeing a consistent doctor'. The GPs in our study were a mix of full- and part-time partners and salaried GPs, and data indicate that they valued and made efforts to provide continuity of care for their patients. Their concern in this regard was, however, that walk-in centres and ports of call other than GP surgeries have the potential to erode continuity of care. Round *et al* (2013) conclude that ‘early diagnosis is the result of the best interaction between patients and their GP' our participants' views echo this assertion, albeit from a different standpoint. Some of the challenges that participants felt they faced also related to recent organisational changes – for example, an increased responsibility for resource management was felt to be at odds with the GP's role as patient advocate.

The strengths of primary care were evident when GPs provided in-depth knowledge of their patient populations and were able to specify which patients were most likely to receive a later diagnosis of cancer. These data highlighted the disparities in health outcomes across socio-economic and socio-cultural groups and individuals, findings that are consistent with the work of others (Forest *et al*, 2013; Nicolson *et al*, 2014). It is right, therefore, that public health initiatives encourage people to contact their GPs if worrying symptoms manifest. It is worth noting, however, that GPs practising in more affluent areas were most likely to make comments regarding increased numbers of consultations, whereas those working in more deprived areas perceived that many of their patients were less affected by public health initiatives. It is also key, therefore, that public health initiatives are targeting ‘hard to reach' areas (National Institute for Health and Clinical Care Excellence (NICE), 2007; Department of Health, 2011), although in this regard it is well to be aware of the arguments others have made that public health campaigns can at times perpetuate health inequalities for some patients in underserved patient populations (Capewell and Graham, 2010; Lorenc *et al*, 2013).

Our findings demonstrate that the burden to GPs of cancer detection is not in the number of patients diagnosed, but in the number of patients they see with potential cancer symptoms who need to be assessed by them and the appropriate action taken. The fact that GPs are able to respond appropriately to their patients is therefore vital, through reassurance, diagnostic investigation before/at the same time as referral and referral routes for non-specific yet suspicious symptoms, all of which require adequate support and resources, as well as opportunities for inter-professional dialogue. Vedsted and Olesen (2011) argue that ‘general practice can provide timely and comprehensive cancer diagnosis when given the proper conditions, for example, diagnostic work-up and referral of patients.' Our findings further substantiate these authors' claims and provide insight from GPs that these would help in the provision of a more robust approach to earlier cancer recognition and referral in primary care.

### Implications for research

Future work with professionals and indeed patients and the public is required to consider the role that professionals could have in addressing the common fear of cancer and how that is related to help-seeking behaviour. In addition, the role of primary care professionals in promoting awareness of cancer symptoms and the cancer screening programmes is worthy of further study.

## Box 2. Symptom recognition and referral by general practitioners

**Public perceptions of cancer**

- Generally speaking the majority of our patients, erm, the big fear is cancer, that's what they see on the telly, that's what they see in the paper … Any symptom, any lump is cancer in our patients … A lot of our consultations revolve around cancer, even though it might be nothing to do medically with cancer. So it's actually very important, but not just from the pure cancer viewpoint. (GP16/M/8)
- There's more a fear of cancer than an awareness of it, so you'll get people coming in with symptoms saying, have I got cancer? And none of the symptoms are suggestive of it. (GP31/M/10)
- I would say I see a patient with bad cancer phobia once a month and, and there's loads of them out there. (GP39/M/8)
- We've got lots of patients who are sort of, erm, cancer phobic; we haven't got many patients who are stroke phobic or heart attack phobic. (GP36/M/7)
- [There is] a generalised fear of cancer … and what you want to do is try and put across that risk without making them too scared. So that's a hard balance to make … We get enough health anxiety without promoting anymore. (GP40/M/8)
- You've got lots of people whose lives are really difficult for all sorts of reasons, and [health] is not prioritised in the same way. (GP24/F/5)
- There's a real fatalistic attitude amongst a lot of people that they sometimes see illness as inevitable because they're seeing it around them, you know, their family smoke, drink heavily, their parents might be having COPD at an early age, they see it as a sort of progressive thing. People round here have very hard lives and I think sometimes that prevention agenda, it's just not so important for them. (GP41/M/1)
- Lower expectations of how long they would even live because some patients are, erm, of, of kind of saying oh I, it's my age and I'm fifty and you go hold on but you're only fifty, you shouldn't be feeling that way so, it's kind of expectations of, of health are lower I think in terms of what, you know, the, how affluent that person is as well, I think is an important part of it. Because perhaps their family history is that they had other family members who maybe had heart attacks in their sixties. So it's expectations, which is very sad. (GP8/M/5)

**Recognition of symptoms**

- You don't want to miss anything and being the gatekeeper is great, erm, but sometimes you tend to find that opening that door is easier than keeping it closed because you're always fearful of missing something. (GP40/M/8)
- Doctors are trained to be pessimists, we always think the worst, so that we don't miss the worst so when I'm talking [to a patient] and saying that there's a risk that could be something related to cancer, that doesn't mean it is cancer, it means I don't want to miss it if it is cancer. (GP2/M/3)
- In any consultation certainly I am always looking to exclude the worst case scenario. (GP35/F/8)
- Sometimes it takes several consultations before you really work out what's going on. (GP23/F/9)
- The skill of General Practice is the fact that we see patients very early in their illness process and it's trying to pick the ones out that are going to not resolve, and these early non-specific symptoms are actually a problem. (GP48/M/8)
- We're trained in a differential diagnosis approach, that means that when somebody comes in with symptoms we think ah, this could be a range of things, it could be something that's self-limiting, something that progresses, it could be something that's extremely serious, it could be something that's serious but is, is treatable, so we're thinking of a range of things and what we do is we, we use time, we use wait, you know, we wait, we see can this resolve on its own. (GP52/F/3)
- Watching and waiting, bit of medicine, see how you get on, you know, a lot of things that we see are self-limiting … given time they will go. (GP22/M/7)
- Most of the cases are going to be benign, self-limiting, erm, not sinister but you've just got to be aware of that things can be nasty and refer on appropriately, so it's just getting that balance between sort of not worrying the patient unnecessarily, but acting promptly when you need to. (GP1/F/4)

**Gatekeeper role**

- Our role is gatekeeper, go between, between patients and the hospital, we're there as an intermediary, an advocate for them. Guide them in the right direction, point them in the right direction, I think that's what GPs role is really. We do look after a lot of chronic things ourselves but otherwise it's assessing people and deciding who needs further intervention, who doesn't. What we can manage, what we can't. (GP23/F/9)
- Secondary care relies on proficient general practice to make sure that that which needs to get through gets through and hopefully we filter out that which doesn't. (GP24/F/4)
- We are the gatekeepers. We are that very first step, so that decision that we made right at the beginning can have massive consequences. (GP18/M/9)
- I think that that gatekeeper role is being eroded by the NHS changes … I just see successive governments not valuing general practice that much and always judging us by … the cases that go wrong. (GP3/M/6)
- If the gatekeeper duty of GPs becomes, you know, more powerful sort of thing, you know, and saying you've got save money, you've got to refer less people, yeah, you know, will that have an impact on, on what we do? (GP7/M/7)

**Referral**

- If somebody comes in with barn door red flag symptoms, you're like yes, this is easy, we've got a lovely [2WW] form, tick all the right boxes and it's quite good because the form kind of makes you, concentrates you on getting the appropriate symptoms, so that's good too. Erm, you put that package together, and off they go and they've got their appointment within two weeks, so for those, the services where we've got that option, and where it's clear cut, it's great. (GP5/F/2)
- Someone comes in coughing up blood, not difficult, er, someone comes in with sort of vague symptoms that turn out to be lymphoma or pancreatic cancer, difficult, you know. (GP39/M/8)
- All my patients who are on the two week wait are seen within two weeks, they're seen the next day. I think it's so popular with the health profession, certainly with GPs, I don't know how secondary care feels, because they're the ones under pressure so they're the ones who've got to keep on top of it, so I don't know how they feel about two week wait but certainly from a GP and patient perspective, it's amazing. (GP27/F/5)

**2WW limitations**

- In general practice, there's always room for that kind of, well gut feeling as well and that you just develop through experience … I think, you know, you can only take those things [guidelines; risk scores] to a certain level, but you've kind of got to use your common sense and experience and your kind of, I'm just worried about this patient, you know, I need to do something here. (GP47/F/10)
- The problem with the two week rule is, you're either two week rule or you're waiting for ages and it's the bit, it's those people in the middle. I think that's the problem, they're the problem patients. (GP11/M/7)
- You've almost got to decide exactly where somebody wants to go. (GP7/M/7)
- From a general practice point of view what you want is you want to refer in, erm, to a place where, erm, a person's going to pick it up on the other side to do general investigations and then streamline. (GP52/F/3)
- You could have [an] ‘I think they've got cancer' two week wait. (GP36/M/7)
- We need a third category – urgently concerned but I don't think it's cancer. (GP24/F/5)
- By the time someone meets the criteria for two week wait symptoms, they've actually got quite progressed cancer by the time they enter the hospital system. (GP22/M/7)

**Strategies to overcome limitations**

- We're quite good as GPs at sidestepping things to get things moving but it would be really useful to have a clear and robust mechanism where we could say, look, this is a real problem because when we, when we come again, across this, we're stuck and all we can do is x when really what we'd like to do is y, and can that be looked at? And if y is thought to be a good idea, can it be sorted, please? Because it, it still seems to me that although we technically have the money and the referral budget, the hospital still block certain things. (GP8/M/5)
- We tick a box [on the 2WW form] even though it's a lie … because sometimes you just think, they've got to be seen, this is ridiculous. (GP23/F/9)
- I probably manipulate the boxes, so if I think that a patient has a high suspicion of cancer I'll probably still send them on a two week form and I put a letter and a note with it to say why, because patients don't always tick boxes. (GP49/F/8)
- Why can't we just say the truth and the other side accept that actually these are vague symptoms, we need to now investigate it. So you almost need like a vague system two week wait pathway, don't you? (GP16/M/8)
- We honestly end up if we're at all worried perhaps abusing the two week wait system slightly when patients do not have Red Flag symptoms, or indeed when the patient's got extreme anxiety. GP39/M/8)
- Make the tests available to me and don't penalise me for using them, so make those scans available for me and don't dis-incentivise me from ordering that test. (GP32/M/3)

**Secondary care relationships**

- In the past you might have rung up the consultants, had a little bit of a chat and he'd say, okay, yes, bring, yeah, ring my secretary, get them put into the clinic in the next couple of weeks, or no, this doesn't need to be seen, do this, this and this, and then I'll see them, and, and you, you had a bit more of a dialogue. (GP11/M/7)
- Twenty years ago you actually wrote a letter, I've a diagnostic problem, and you would write to a named consultant who'd have a look at that letter and think, yeah, I know what that problem is, whereas now you, it's, you just book an appointment and you, it's almost like we've got to decide where to book it and we don't know where they've got to go and they get stuck in a clinic. (GP7/M/7)
- You know they're not going to be seen for eighteen weeks unless you do something, but they don't fit the two week pathway, so what do you do? … Ringing the secretary's always a good one and saying, you know, can we have this one like at the front of the queue or can you just run it by the consultant? You've almost got to play those individually just to get the best deal for your patient. (GP14/F/10)
- Anything that brings primary and secondary care together can only be good for the patient. So whatever policy gets developed, how does it develop primary care and how does it bring primary and secondary care closer. (GP52/F/3)

## Box 3. Fragmentation of NHS services and care

**Continuity of care**

- The main thing I personally think is having that rapport with your patients, as a practice, where they feel they can approach you early about anything and then having that kind of diagnostic sieve in your mind of, you know, that's suspicious, let's refer that and then having that relationship with secondary care and then following patients up afterwards and discussing them so you can learn things from it. (GP1/F/4)
- I don't like these walk-in centres. I don't think they serve anything apart from for adding yet another tier of unlinked up care. (GP24/F/5)
- Open access to walk-in centres, A&E etcetera is a bad idea, erm, because, you know, they, they don't have the whole picture, they'll commonly tell patients they need investigations and things like that and we, we're a bit of a loser and it's very financially, a very bad idea and it means … limited resources don't get used properly. (GP39/M/8)
- I'm a strong believer that the National Health Service does deliver good care to the majority of patients and … it is being eroded, erm, because patients do have access into the NHS through different portals now, you've got walk-in centres, you've got Darzi Centres in some areas that are completely unnecessary … every area had to have a, a general practice that was open eight until eight, seven days a week, where patients can just walk-in and see a GP. Now I don't argue with that at all in areas of need, yeah, Central London, Birmingham, Manchester, whatever but you don't need one [here], where you've got eleven good practices with pretty good access. (GP3/M/6)
- Continuity of care is, you know, something that would be helpful [and] it is generally reasonably feasible. I mean it, it's not an absolute given that you'll get that because so many of us are part-time, I mean I only, I work three days a week as do two of my colleagues, er, we have salaried doctors, one of whom is only here for two days a week, you know, there's, so it, it can be awkward, erm, but actually it, with a bit of planning it's possible, erm, to at least limit the number of people that you're seeing for a given problem … before the new GP contract a few years ago, we used to insist on it, you know, you, you saw your doctor, which meant that people became very well known to each other and, er, and I think it did help then to gauge when, when a situation had changed significantly and needed more investigation. So I think that, that's been an issue in investigation of cancer. (GP14/F/10)
- [The patient] had come to us originally but then he'd gone to a walk-in centre, then he'd gone to casualty, then he'd gone back to the walk-in centre then he came back to us, so, so you're almost at that point thinking that's only your, our second visit, whereas really it was about his fourth or fifth visit, now somebody coming back five times, here, for the same problem, you would be thinking something weird is going on here, we've got to get this sorted, but it almost didn't ring the same alarm bells because we didn't really know about the other stuff. (GP5/F/2)

**Organisational change**

- I'm not optimistic that new NHS structures are going to have as big an impact, erm, on, erm, the way in which we handle diseases and groups of people in the community as perhaps they might do … this is like, you know, changing gears without any, erm, clutch, I mean it's really grinding the cogs quite badly. Erm, and I think, you know, suddenly to find all this has been deposited in our lap to do, it's a great challenge. (GP2/M/3)
- [CCGs will] take an awful lot of experienced GPs away from their desks, that is definitely one thing it's going to do. So that's going to affect cancer diagnosis isn't it? Because people are going to have to rely on locums or younger GPs filling in, because I think with commissioning and these consortiums and things, that's taken up tons of GP time, so on a purely practical level I think it will have a negative effect on the running of general practices full stop … cancer and everything else. (GP5/F/2)
- I fear the day that we make the choices of who gets what and you have to do it on a face to face basis … trying to rationalise that as a patient advocate and a care provider is very difficult, you need somebody to be able to say, you're not having this because of this, and if it ends up being the GP then that is very difficult to be able to justify to the patient and maintain a decent relationship. You've got to have a bad guy and if the GP is the bad guy then they'll stop coming to see their GP and I think that'll be very difficult to deal with, erm, because they've got to be able to trust you, and if they don't then they aren't going to come. (GP40/M/8)
- Now with the pressures of, you know, the CCGs and money, that referral threshold's probably getting higher and higher because referrals all cost money and more investigations cost money so that may be a pressure for some, although I feel it shouldn't be and again if suddenly finances get so tight that, or limited to what we can do, then even if you didn't want to, you'd probably subconsciously think actually, erm, should I, should I not send them for this scan? (GP34/M/1)
- I don't know if it becomes cost limited, you know, if every time we order an investigation how that will impinge on us. So, you know, if you have, if you had to decide each time whether it was worth [investigation]. (GP23/F/9)
- Asking me to do all this admin then I can't see patients. (GP32/M/3)

## Box 4. Primary Care/Public Health interface

**Screening**

- You've got to have a test that works and you've got to have, and you've got to be able to do something about it when you've actually picked something up otherwise, you know, maybe it's better not to go screening. (GP33/M/3)
- Cervical is ongoing, yes, yeah, and we still have to chase people, we're still trying to get eighty percent of our population screened is hard work, people don't want to have it done. For a whole host of reasons, erm, breast screening, breast screening, you know, I, it's, is it good, is it bad? You know, it is not necessarily and there's no doubt there's a vast, over diagnosis, over treatment, erm, of it. Prostate screening, the jury's definitely out, definitely again, er, but it's a big problem we have, again media driven, you know, it's being covered more and more and quite regularly have conversations with fifty or sixty year old gents coming in and wanting their prostate cancer blood test. (GP48/M/8)
- We don't have evidence yet, of the impact of mammography screening we got initially great reports, there now seems to be an element of cynicism being poured over the benefit of that. Before I was really bullying people into going for the screening … I mean people would come and say, you know, should I go? You would say yes, yes, it's, it's recommended, but now. (GP7/M/7)
- Why does bowel cancer miss so many? Because it's centrally run. If it was our practice that was chasing our patients for bowel screening, we'd have a higher uptake, I've no doubt about that … the trouble is of course that that process involves funding. (GP16/M/8)
- They've had a mammogram and they're told you don't need another one for five years and then, you know, maybe do get cancer in the interim time and think well what was that test about? That's quite hard for people to, erm, sort of get their heads round in a way, being told we've done that test and it's fine and we'll do it again in five years' time and I think they think a year later how, how can I have cancer or potentially have cancer when that test was fine? Bit tricky. (GP38/M/8)
- The only one I had with a positive [bowel] screening didn't want to go for, for testing, so I just had to speak to him about what he could be missing and ignoring and he chose not to go for a colonoscopy. I think he was terrified actually; he knew what he was ignoring. There's a difference to doing the test in your own home to a colonoscopy. (GP25/F/9)

**Awareness raising initiatives**

- Public education's very good, very important. Erm, it'd be interesting to know whether referrals increase in response to any campaigns, erm, I'm not, from my own experience I'm not aware that it has. (GP24/F/5)
- By and large they are a waste of time, because all the research shows, that I'm aware of, is that patients, I call them patients, the population, people do not change their living activities until they perceive a problem that requires them to change. The vast majority of people do not heed health promotion advice, i.e., if you continue to do this in ten years' time you will develop this, ten years' time is in the future, they don't bother changing. (GP48/M/8)
- Sometimes you feel there's too many patients coming just for this media thing but then you think looking back maybe it's good, maybe that is telling them, I don't have to necessarily scan everybody but at least I'm happy that, you know, they are realising that, I need to check do I have this … of course it can be time consuming, in terms of the consultation, of course it increases on us. (GP10/M/4)
- It's about sowing the seed isn't it? And it's about, erm, raising awareness generally for, for years to come, so I think as, even just a spot check may not be the best way of assessing the impact on that. (GP55/F/9)
- I don't think I've got any personal experience of, of people necessarily saying oh I saw the poster outside and have, you know, want to discuss this problem but, erm, it'd be interesting to know whether it has worked. (GP8/M/5)
- By and large I think they're very good really, I mean raising people's awareness of symptoms and that sort of thing, whether it'll actually translate into earlier diagnosis I'm not so sure because I don't know how many more people will attend because of it really, because unfortunately it, for a lot of people health is not something that they think about it until it actually fails them … We're trying to identify those people who are at risk of cancer. Now, which it's not like a needle in a haystack, because it's a bit more common than that, but the danger is you don't make the needle bigger, you make the haystack bigger, because more people come and they've nothing wrong with them. (GP36/M/7)
- There's a pyramid of work, a lot of work for a smaller outcome than you might imagine. Whether that turns out to be worthwhile or not I can't tell you, you'll have to ask the epidemiologist that in ten years' time. (GP33/M/3)
- It gets through to the worried well, does it get through to people that really have the cancer? (GP25/F/9)
- As part of the safety netting, er, we, we use that kind of, er, awareness anyway, so for example if, if a lady comes with the three weeks cough, we explain to them, if you have a cancer, these are the possible other symptoms what would expect you to have, and then if, if you have any of these symptoms we want to see you straight back. (GP9/M/7)
- I can see that, that there's going to be, a number of people who will come inappropriately early as a result of it and you've got to try and work that out as a GP from people who are coming with, with something genuine that are presenting a little bit earlier, and the earlier you present the harder it is for us to make the right diagnosis, and the slight concern is that if you come too early you might then get put off coming back again. (GP33/M/6)
- There's a danger that if we get so many people coming in saying oh I've got, you know, they, I've been seeing the advert and my poo's changed a bit, then we'll just get, you know, the danger that we will develop a negative response to that, think ah not another one, you know, you will then miss the one that really is, yeah, I think people haven't thought about, that's, there is a big danger. (GP39/M/8)

**GP role in raising awareness**

- Unfortunately it tends to be, you know, the kind of middle class, erm, professional people who are, who are, you know, more demanding generally … The patients I see, the ones who push for referral, it's not the, you know, Bengali man in his seventies who maybe only speaks a little bit of English. (GP26/F/4)
- Patients who lived in our most deprived postcodes they are more likely to have cancers, they're often also less likely to come to the doctor for a check-up, whereas someone in West End of [town] is more likely to, oh this is free, I'll go and have it done. (GP3/M/6)
- It's the usual, you know, people that are poor, people whose English isn't their first language, people who are fatalistic. People who, erm, you know, don't want to bother their doctor, people who, er, come, other people, people that come too often, you know, ones that are here every, every week and, you know, every week you sort of think oh here they are again, must get on with them and invariably one, is something is wrong with them and you missed it. I think that's the other people that I think that we struggle with. (GP54/M/
- You can only diagnose it if people come to see you. (GP12/F/7)
- Apart from literally going through a systemic enquiry every, every time somebody comes in, you know, you've got to be realistic about what you can do haven't you? Yeah, but we can certainly support it [campaigns]. (GP43/M/8)
- I think if we were clear of what we were trying to do and how to do it then we would be very happy to go with, along with a campaign, where we are at the moment doesn't feel as though this practice is about to mount single-handedly any sort of proactive checks for our patients. (GP15/F/3)
- To be quite honest I've got enough to do treating the sick … I haven't got time to have a public role, anyway I'm not paid for a public health job, it's not my job. (GP39/M/8)
- I don't think we've got the, the time or the, or, or the capacity to, you know, we're getting busier and busier and to put an educational role on top of that I think it would be difficult, yeah. (GP31/M/10)
- We make sure that our waiting room is not cluttered but has the relevant important posters, while they're waiting to see the, you know, the nurse or whatever, certain things might ooh actually, you know, that, that's relevant to me, so it's just trying to kind of optimise every, every opportunity you might have for patient contact to sort of educate them really. (GP1/F/4)
- I'll chuck preventative things into, into the consultations, smoking, alcohol, weight, erm, certainly where I practice is, is always relevant, erm. (GP33/M/3)
- We've got a huge, huge role in it really, but it's, it's prevention, we don't have much luck preventing cancer do we? It is just the, the high risk activities really … I usually say you've got to find your own mental reason, you know, do you want to be there for you, your granddaughter's wedding? You know, and what are you aiming for and, you know, who do not want to turn out like and who do you, who do you want to avoid this happening to you? It's got to be very emotive, personal to them to help them stop [smoking], they won't stop just being told by a picture or a word on a packet. Me telling them's not going to make a huge difference. (GP25/F/9)
- More of what we do is about health promotion so I'd see more of myself a role about behaviour modification, behavioural change, which helps reduce someone's risk of developing cancer, I can't say I've ever discussed what symptoms, you know, to be alerted for, it doesn't naturally flow into what you're doing and I, I would see a much bigger role about, about behavioural change. [...] And also that cancer is only a small part of what's in those things, and they cause a lot of other morbidity and illness as well. (GP41/M/1)
- If someone came to me and said, you know, would you come along and give a talk to a, you know, a group of people to, to raise awareness of cancer if I felt able to I would, so I think we do have roles in that respect. (GP3/M/6)
