# Supplementary material for: Cancer detection in primary care: insights from general practitioners
Source: Br J Cancer. 2015 Mar 3;112(Suppl 1):S41–9. doi: 10.1038/bjc.2015.41 (PMC4385975; doi:10.1038/bjc.2015.41)
Supplement: Supplementary Information [file bjc201541x1.doc]

**TOPIC GUIDE**

**Introduction:** There has been a lot of publicity in recent years about the importance of early diagnosis of cancer being important – we are interested to explore GPs views of this and their view of the role of general practice with respect to cancer awareness/detection.

**General**

Where do you think cancer diagnosis fits in terms of all your competing priorities as a practice?

**GP role in early detection**

- Challenges faced by GPs in the recognition of cancer symptoms
- Different issues for common and rare cancers (How/what?)
- Particular cases of cancer diagnoses in your practice that you have learnt from - explore in detail.
- Issues with local referral systems/protocols
- Any system changes in practice to address delays in recognition and referral
- Role of decision support tools - and if so what kinds of decision support tools would be useful?
- What else could enhance GPs ability to recognise cancer symptoms?
- Are there groups of patients who are less likely to receive early diagnosis – and if so, who and why does this happen?
- Understanding of their own gate-keeping role within NHS and how this impacts on early cancer diagnosis

**Role of primary care with respect to cancer awareness**

- Views on how aware patients/the public are of cancer symptoms
- Which symptoms there is greater awareness of, which less
  - Does this vary depending on demography, age, education etc?
- Opportunities for GPs and other primary care professionals to engage with patients about potential cancer symptoms?
  - Are there particular sorts of patients they should be engaging with?
- What is the role of general practice in this wider public health agenda?

**Role of GPs with respect to cancer screening**

- Understanding of developments of screening programmes
- Reasons for lack of uptake
- Potential for primary care role in enhancing uptake of screening
- Opinions about targeted screening- e.g. screening of high risk individuals?

**Policy issues**

- What policy issues have impacted on cancer diagnosis in recent years and in what ways? (prompts: referral guidelines, 2 week wait, choose and book, national cancer audit, practice profiles)
- The key things policy makers could do to make cancer diagnosis in general practice better
- The role for QOF (if any)
- The role for the RCGP (if any)
- What is the potential for the NHS changes to impact on cancer diagnosis?
