# Supplementary material for: Cancer detection in primary care: insights from general practitioners
Source: Br J Cancer. 2015 Mar 3;112(Suppl 1):S41–9. doi: 10.1038/bjc.2015.41 (PMC4385975; doi:10.1038/bjc.2015.41)
Supplement: Supplementary Information [file bjc201541x2.doc]

**GP and practice demographics**

| **GP** | **Gender** | **Pseudonym** | **Years practising*** | **Patient numbers ǂ** | **Deprivation scoreǂ** | **Ethnicity Estimate ǂ** |
| --- | --- | --- | --- | --- | --- | --- |
| 1. 1 | Female | GP1/F/4 | 6 | 1700 | 4 | 98% white British; 2% minority ethnic groups |
| 1. 2 | Male | GP2/M/3 | 25 | 3200 | 3 | 99% white British; 1% minority ethnic groups |
|  | Male | GP3/M/6 | 26 | 10,000 | 6 | 95% white British; 5% minority ethnic groups |
|  | Male | GP4/M/8 | 14 | 5700 | 8 | 96% white British; 4% minority ethnic groups |
|  | Female | GP5/F/2 | 20 | 10,200 | 2 | Insufficient data |
|  | Male | GP6/M/2 | 21 | 9900 | 2 | 98% white British; 2% minority ethnic groups |
|  | Male | GP7/M/7 | 24 | 5000 | 8 | 96% white British; 4% minority ethnic groups |
|  | Male | GP8/M/5 | 13 | 7200 | 5 | 98% white British; 2% minority ethnic groups |
|  | Male | GP9/M/7 | 5 | 6300 | 7 | 98% white British; 2% minority ethnic groups |
|  | Male | GP10/M/4 | 8 | 2300 | 4 | 50% white British; 50% minority ethnic groups |
|  | Male | GP11/M/7 | 21 | 7200 | 7 | 98% white British; 2% minority ethnic groups |
|  | Female | GP12/F/7 | 14 | 7200 | 7 | 98% white British; 2% minority ethnic groups |
|  | Male | GP13/M/5 | 3 | 7000 | 5 | 93% white British; 7% minority ethnic groups |
|  | Female | GP14/F/10 | 19 | 5000 | 10 | 93% white British; 7% minority ethnic groups |
|  | Female | GP15/F/3 | 25 | 7700 | 3 | 91% white British; 9% minority ethnic groups |
|  | Male | GP16/M/8 | 7 | 6400 | 8 | 96% white British; 4% minority ethnic groups |
|  | Male | GP17/M/9 | 26 | 7300 | 9 | 99% white British; 1% minority ethnic groups |
|  | Male | GP18/M/9 | 9 | 9200 | 9 | 98% white British; 2% minority ethnic groups |
|  | Male | GP19/M/9 | 18 | 9200 | 9 | 98% white British; 2% minority ethnic groups |
|  | Male | GP20/M/3 | 13 | 8700 | 3 | 96% white British; 4% minority ethnic groups |
|  | Female | GP21/F/6 | 7 | 36,000 | 6 | 57% white British; 43% minority ethnic groups |
|  | Male | GP22/M/7 | 12 | 4400 | 7 | 99% white British; 1% minority ethnic groups |
|  | Female | GP23/F/9 | 22 | 7300 | 9 | 99% white British; 1% minority ethnic groups |
|  | Female | GP24/F/5 | 26 | 7100 | 5 | 83% white British; 17% minority ethnic groups |
|  | Female | GP25/F/9 | 8 | 9600 | 9 | 84% white British; 16% minority ethnic groups |
|  | Female | GP26/F/4 | 23 | 12,300 | 4 | 33% white British; 67% minority ethnic groups |
|  | Female | GP27/F/5 | 16 | 8600 | 5 | 41% white British; 59% minority ethnic groups |
|  | Female | GP28/F/10 | 25 | 12,500 | 10 | 92% white British; 8% minority ethnic groups |
|  | Female | GP29/F/10 | 11 | 12,500 | 10 | 92% white British; 8% minority ethnic groups |
|  | Male | GP30/M/10 | 22 | 12,500 | 10 | 92% white British; 8% minority ethnic groups |
|  | Male | GP31/M/10 | 24 | 12,500 | 10 | 92% white British; 8% minority ethnic groups |
|  | Male | GP32/M/3 | 5 | 7500 | 3 | 25% white British; 1% minority ethnic groups |
|  | Male | GP33/M/6 | 25 | 15,500 | 6 | 99% white British; 75% minority ethnic groups |
|  | Male | GP34/M/1 | 7 | 13,000 | 1 | 30% white British; 70% minority ethnic groups |
|  | Female | GP35/F/8 | 24 | 14,600 | 8 | 97% white British; 3% minority ethnic groups |
|  | Male | GP36/M/7 | 26 | 14,500 | 7 | 98% British; 2% minority ethnic groups |
|  | Female | GP37/F/4 | 2 | 1800 | 4 | 98% white British; 2% minority ethnic groups |
|  | Female | GP38/F/8 | 10 | 13,100 | 8 | 99% white British; 1% minority ethnic groups |
|  | Male | GP39/M/8 | 26 | 6500 | 8 | 98% white British; 2% minority ethnic groups |
|  | Male | GP40/M/8 | 5 | 5800 | 8 | 99% white British; 1% minority ethnic groups |
|  | Male | GP41/M/1 | 11 | 5900 | 1 | 91% white British; 9% minority ethnic groups |
|  | Female | GP42/F/8 | 12 | 9800 | 8 | 99% white British; 1% minority ethnic groups |
|  | Male | GP43/M/8 | 16 | 9800 | 8 | 99% white British; 1% minority ethnic groups |
|  | Female | GP44/F/8 | 22 | 9800 | 8 | 99% white British; 1% minority ethnic groups |
|  | Female | GP45/F/1 | 1 | 2000 | 1 | 96% white British; 4% minority ethnic groups |
|  | Male | GP46/M/10 | 26 | 3300 | 10 | 99% white British; 1% minority ethnic groups |
|  | Female | GP47/F/10 | 15 | 3300 | 10 | 99% white British; 1% minority ethnic groups |
|  | Male | GP48/M/8 | 22 | 14,000 | 8 | 98% white British; 2% minority ethnic groups |
|  | Female | GP49/F/8 | 20 | 14,000 | 8 | 98% white British; 2% minority ethnic groups |
|  | Female | GP50/F/6 | 9 | 2300 | 6 | 50% white British; 50% minority ethnic groups |
|  | Male | GP51/M/9 | 5 | 6000 | 9 | 82% white British; 19% minority ethnic groups |
|  | Female | GP52/F/3 | 22 | 4000 | 3 | 35% white British; 65% minority ethnic groups |
|  | Male | GP53/M/2 | 22 | 7400 | 2 | 36% white British; 64% minority ethnic groups |
|  | Male | GP54/M/3 | 18 | 7800 | 3 | 34% white British; 66% minority ethnic groups |
|  | Female | GP55/F/9 | 21 | 4500 | 9 | 99% white British; 1% minority ethnic groups |

**(Sources: *Interview transcripts; ǂ National General Practice Profiles** [**http://fingertips.phe.org.uk/profile/general-practice**](http://fingertips.phe.org.uk/profile/general-practice)**)**
