# Supplementary material for: Cancer detection in primary care: insights from general practitioners
Source: Br J Cancer. 2015 Mar 3;112(Suppl 1):S41–9. doi: 10.1038/bjc.2015.41 (PMC4385975; doi:10.1038/bjc.2015.41)
Supplement: Supplementary Information [file bjc201541x3.doc]

Caption for SOM 1

Topic Guide

SOM 2 caption

GP and practice demographics
